# Association of urinary dipeptidyl peptidase 4 activity with clinical outcomes in people with chronic kidney disease

**DOI:** 10.1038/s41598-025-06395-x

**Published:** 2025-07-02

**Authors:** Acaris Benetti, Joao Carlos Ribeiro-Silva, Luz M. Gómez, Caio A. M. Tavares, Isabela J. Bensenor, Paulo A. Lotufo, Silvia M. O. Titan, Adriana C. C. Girardi

**Affiliations:** 1 Laboratório de Genética e Cardiologia Molecular, Faculdade de Medicina, Instituto do Coração (InCor), Hospital das Clinicas HCFMUSP, Universidade de São Paulo, Avenida Dr. Enéas de Carvalho Aguiar, 44-Bloco II 10° Andar, São Paulo, SP 05403-900 Brazil; 2 State University of New York (SUNY) Upstate Medical University, Syracuse, NY USA; 3 Departamento de Estatística, Universidade Federal de Pernambuco, Recife, PE Brazil; 4 Unidade de Geriatria, Faculdade de Medicina, Instituto do Coração (InCor), Hospital das Clínicas HCFMUSP, Universidade de São Paulo, São Paulo, SP Brazil; 5 Hospital Israelita Albert Einstein, São Paulo, SP Brazil; 6 Hospital Universitario, Universidade de Sao Paulo, São Paulo, Brazil; 7 Divisão de Nefrologia, Faculdade de Medicina, Hospital das Clínicas HCFMUSP, Universidade de São Paulo, São Paulo, SP Brazil; 8 Nephrology and Hypertension Division, Mayo Clinic, Rochester, MN USA

**Keywords:** Physiology, Cardiology, Diseases, Nephrology, Pathogenesis, Risk factors

## Abstract

Experimental studies have shown that urinary dipeptidyl peptidase 4 (uDPP4), unlike serum DPP4 (sDPP4) activity, correlates with proteinuria, serum creatinine, and left ventricular (LV) hypertrophy in chronic kidney disease (CKD) models, suggesting a potential role for uDPP4 in CKD progression. This study examined the relationship of uDPP4 and sDPP4 activities with renal, cardiovascular, and metabolic markers, along with mortality and initiation of kidney replacement therapy (KRT) events in individuals with CKD. DPP4 activity was measured in the urine and serum of 426 participants from the Brazilian CKD cohort, PROGREDIR. Participants were stratified into tertiles based on uDPP4 and sDPP4 activities. Multivariable linear regression, Kaplan–Meier analysis, Cox hazards, and competing risk models (cause-specific and Fine–Gray) were used. uDPP4 activity was positively associated with albuminuria, urinary retinol-binding protein 4, LV mass, and type 2 diabetes but inversely associated with body mass index and use of renin-angiotensin system blockers. In contrast, sDPP4 activity correlated only with age and biological sex. Higher uDPP4 activity was associated with a higher incidence rate of all-cause mortality (p < 0.0001). Participants in the second and third uDPP4 activity tertiles had greater mortality risk compared to the lowest tertile (HR 2.03, 95% CI 1.36–3.04 and HR 2.48, 95% CI 1.67–3.67, respectively), even after controlling for potential confounders. No independent association was found between sDPP4 activity and mortality or initiation of KRT. These findings support uDPP4’s involvement in CKD progression and its association with increased mortality risk in individuals with CKD.

## Introduction

Dipeptidyl peptidase 4 (DPP4), or CD26, is a 110 kDa multifunctional protein involved in diverse physiological and pathophysiological processes^1^. It cleaves the N-terminal dipeptide from peptides with proline or alanine in the second position^2^, including glucagon-like peptide-1 (GLP-1)^3,4^, glucose-dependent insulinotropic polypeptide (GIP)^4^, peptide tyrosine-tyrosine (PYY)^5^, neuropeptide Y (NPY)^5^ and stromal-derived factor 1 (SDF-1)^6^. Beyond its catalytic role, DPP4 interacts with a variety of proteins, such as adenosine deaminase^7^, the sodium-hydrogen-3 exchanger (NHE3)^8^, fibronectin^9^, collagen^10^, the C-X-C chemokine receptor type 4 (CXCR4)^11^, and the CD45 tyrosine phosphatase^12^. These interactions facilitate various biological processes, including signal transduction, inflammation, natriuresis, and cellular interactions with the extracellular matrix.

DPP4 is expressed on the surface of kidney, intestine, and liver epithelial cells and in adipocytes, endothelial cells, fibroblasts, lymphocytes, and smooth muscle cells^1^. The highest abundance of DPP4 is found in the kidney, specifically as one of the major proteins in the apical membrane of the proximal tubules^2,8^. Human DPP4 expression and enzymatic activity are also observed in the glomerulus, mainly in podocytes, but only under pathological renal conditions, not in healthy kidneys^13^. A 90 kDa truncated form of DPP4 is generated through proteolytic cleavage (shedding) by matrix metalloproteinases, releasing a soluble, enzymatically active form of DPP4 into body fluids^1,14^.

Elevated serum DPP4 levels have been associated with obesity^15^, hyperinsulinemia^16,17^, coronary artery disease^18^, and heart failure^19^. In the 5/6 nephrectomy rat model of chronic kidney disease (CKD), we demonstrated that urinary DPP4 (uDPP4) abundance and activity increase with disease progression, while serum DPP4 (sDPP4) remains unchanged^20^. In this model, DPP4 inhibition attenuated glomerulosclerosis and tubulointerstitial injury, thereby slowing disease progression^20,21^. It also improved CKD-associated cardiac dysfunction by reducing cardiac hypertrophy and fibrosis, lowering blood pressure, and enhancing diastolic function through DPP4 inhibition^21,22^. Moreover, DPP4 inhibition has proven beneficial in experimental models of diabetes^23^ and obesity-associated renal dysfunction^24,25^, suggesting that DPP4 plays a role in a common mechanism underlying the development and progression of kidney disease.

In humans, DPP4 inhibition was associated with reduced albuminuria in the Saxagliptin and Cardiovascular Outcomes in Patients with Type 2 Diabetes (SAVOR-TIMI 53) trial^26^. In the Cardiovascular and Renal Microvascular Outcome Study with Linagliptin (CARMELINA) trial^27^, linagliptin prevented the progression from microalbuminuria to macroalbuminuria in patients with type 2 diabetes (T2D) who were at high risk of cardiovascular and kidney events. Another study showed that the use of DPP4 inhibitors was associated with a reduced risk of acute kidney disease in diabetic patients and a lower risk of dialysis requirement during acute kidney disease events^28^. However, the association between uDPP4 and sDPP4 activities and clinical outcomes is yet to be fully explored.

Given the experimental evidence implicating DPP4 in the pathogenesis of CKD and the demonstrated clinical benefits of DPP4 inhibitors, this study investigated whether uDPP4 activity is associated with an increased risk of CKD progression and mortality in individuals with CKD.

## Results

### Descriptive data and cross-sectional analysis

Among the 454 participants from the PROGREDIR cohort, 426 were included in the present analysis. Measurements of uDPP4 and sDPP4 activities revealed a non-Gaussian distribution with notable positive skewness and kurtosis, as illustrated in Supplementary Fig. S1. uDPP4 activity ranged from 0 to 308 nmol/min/g creatinine, with a median of 7 nmol/min/g (IQR 1–37 nmol/min/g) (Supplementary Fig. S1A). sDPP4 activity ranged from 15 to 96 nmol/min/mL, with a median of 36 nmol/min/mL (IQR 31–44 nmol/min/mL) (Supplementary Fig. S1B).

Table 1 summarizes the descriptive characteristics of the 426 participants. For some variables, data were unavailable for all individuals, and the corresponding sample sizes are noted. The final sample included 272 men (64%) with a mean age of 67 years (SD, 12). Most participants were at CKD stage 3b (37%); 242 (57%) had diabetes, 383 (90%) had hypertension, and 259 (61%) were receiving RAS blockers. Table 1 delineates variables according to the tertiles of uDPP4 and sDPP4. Increasing tertiles of uDPP4 were significantly associated with higher systolic blood pressure (SBP), left ventricular (LV) mass, albuminuria, and urinary RBP4 (uRBP4), alongside decreasing values of BMI, eGFR, and use of RAS blockers. Conversely, increasing tertiles of sDPP4 activity correlated with higher serum glucose, glycated hemoglobin, total cholesterol, LDL, and albuminuria, as well as decreased age and biological sex (higher proportion of women in the highest tertile).

**Table 1:** Baseline demographics and clinical characteristics of PROGREDIR cohort participants.

	All participants	uDPP4 (nmol/min/g creatinine)				sDPP4 (nmol/mL/min)			
		Tertile 1	Tertile 2	Tertile 3	p for trend	Tertile 1	Tertile 2	Tertile 3	p for trend
		(≤ 3.3)	(3.3–17.7)	(≥ 17.7)		(≤ 33.0)	(33.0–41.3)	(≥ 41.3)	
	n = 426	n = 141	n = 140	n = 145		n = 142	n = 140	n = 144	
Age, years (SD)	67 (12)	65 (12)	67 (12)	68 (11)	0.23	70 (12)	66 (12)	64 (12)	< 0.001
Male sex, n (%)	272 (64)	92 (65)	94 (67)	86 (59)	0.36	103 (73)	91 (66)	78 (53)	0.002
Hypertension, n (%)	383 (90)	125 (89)	127 (91)	131 (91)	0.83	134 (94)	122 (88)	127 (87)	0.09
SBP, mmHg (IQR)	140 (32)	132 (23)	137 (27)	145 (37)	0.006	134 (32)	138 (36)	140 (32)	0.28
DBP, mmHg (IQR)	75 (17)	76 (14)	74 (18)	75 (17)	0.94	74 (18)	75 (15)	77 (16)	0.17
LV mass, g/m^2^ (IQR)	126 (45)	116 (38)	128 (46)	134 (46)	< 0.001	128 (52)	125 (46)	123 (37)	0.16
Ejection fraction (SD)	0.61 (0.13)	0.60 (0.14)	0.62 (0.13)	0.62 (0.13)	0.36	0.6 (0.14)	0.6 (0.13)	0.6 (0.13)	0.36
Acute myocardial infarction, n (%)	152 (38)	47 (31)	50 (33)	55 (36)	0.59	47 (33)	48 (34)	47 (33)	0.78
Smoking (current or previous), n (%)	259 (61)	87 (33)	90 (35)	82 (32)	0.25	86 (33)	87 (34)	86 (33)	0.90
Type 2 diabetes, n (%)	242 (57)	73 (51)	79 (56)	90 (62)	0.21	79 (56)	73 (53)	90 (62)	0.31
Fasting serum glucose, mg/dL (IQR)	104 (32)	103 (25)	106 (29)	104 (41)	0.18	102 (23)	104 (31)	109 (39)	0.02
Hb1Ac, % (IQR)	6.2 (1.4)	6.2 (1.1)	6.3 (1.7)	6.2 (1.6)	0.55	6.1 (1.3)	6.1 (1.4)	6.3 (2.3)	0.03
Body mass index, kg/m^2^ (SD)	30 (6)	30 (6)	30 (6)	28 (5)	0.02	29 (5)	30 (6)	29 (6)	0.17
Total cholesterol, mg/dL (SD)	169 (40)	167 (41)	170 (39)	169 (41)	0.85	159 (35)	166 (40)	180 (42)	< 0.001
LDL-cholesterol, mg/dL (SD)	91 (32)	91 (32)	92 (30)	91 (34)	0.92	86 (28)	91 (32)	96 (36)	0.03
HDL-cholesterol, mg/dL (SD)	46 (14)	45 (17)	45 (11)	48 (14)	0.29	46 (17)	44 (13)	48 (13)	0.21
Serum creatinine, mg/dL (IQR)	1.7 (0.7)	1.7 (0.6)	1.6 (0.6)	1.7 (1.0)	0.17	1.6 (0.7)	1.7 (0.8)	1.7 (0.8)	0.24
eGFR, mL/min/1.73 m^2^ (SD)	41 (16)	42 (15)	43 (16)	38 (16)	0.05	43 (15)	41 (16)	40 (16)	0.25
CKD stages, n (%)					0.19				0.16
1 + 2	52 (12)	16 (12)	21 (15)	15 (10)		17 (12)	19 (14)	16 (11)	
3a	107 (25)	40 (28)	37 (26)	30 (21)		46 (32)	28 (20)	33 (23)	
3b	156 (37)	55 (39)	50 (36)	51 (35)		52 (37)	50 (36)	54 (37)	
4 + 5	111 (26)	30 (21)	32 (23)	49 (34)		27 (19)	41 (30)	43 (29)	
Urinary ACR, mg/g (IQR)	85 (622)	27 (182)	102 (594)	272 (1124)	< 0.001	55.5 (169)	128.5 (690)	103 (895)	0.02
Urinary RBP4, mg/g (IQR)	0.29 (1.27)	0.23 (0.83)	0.19 (0.88)	0.46 (3.19)	< 0.001	0.18 (0.78)	0.31 (1.74)	0.29 (1.12)	0.08
FE Phosphate, %; (IQR)	25 (16)	25 (15)	23 (16)	25 (17)	0.65	24 (14)	23 (19)	25 (16)	0.69
Use of RAS blockers, n (%)	259 (63)	91 (65)	95 (68)	73 (50)	0.06	92 (65)	78 (57)	89 (61)	0.37

Data available for: LV mass, *n* = 412; BMI, *n* = 425; uRBP4, *n* = 415; FE Pi, *n* = 388; use of RAS blockers, *n* = 411. Tukey’s and Friedman’s post hoc tests were used for normally and non-normally distributed variables, respectively. Chi-squared test was used for categorical comparisons.

*ACR* albumin creatinine ratio, *CKD* chronic kidney disease, *DBP* diastolic blood pressure, *eGFR* estimated glomerular filtration rate, *FE* fraction of excretion, *Hb1Ac* glycated hemoglobin, *IQR* interquartile range, *LV* left ventricle, *RAS* Renin-Angiotensin System, *RBP4* retinol-binding protein 4, *SBP* systolic blood pressure, *SD* standard deviation.

Table 2 and Supplementary Figure S2 present the linear univariable models and correlation coefficients of clinical variables at baseline on uDPP4 and sDPP4. SBP, LV mass, T2D, BMI, eGFR, albuminuria, uRBP4, and the use of RAS blockers were associated with uDPP4, while SBP, age, biological sex, albuminuria, and uRBP4 were correlated with sDPP4. In the multivariable model, LV mass, T2D, BMI, albuminuria, uRBP4, and the use of RAS blockers remained independently associated with uDPP4, whereas only age and biological sex remained associated with sDPP4 activity (Table 2).

**Table 2:** Association of uDPP4 and sDPP4 with age, biological sex and renal, cardiovascular, and metabolic function markers.

	Univariate			
	(log) uDPP4		sDPP4 (nmol/mL/min)	
	n = 426		n = 426	
	β (95% CI)	p-value	β (95% CI)	p-value
Age, years	0.02 (− 0.003 to 0.04)	0.09	− 0.25 (− 0.33 to 0.17)	< 0.0001
Male sex	− 0.34 (− 0.85 to 0.17)	0.19	− 3.83 (− 5.92 to − 1.74)	< 0.001
SBP, mmHg	0.02 (0.01 to 0.03)	< 0.0001	0.04 (0.003 to 0.09)	0.03
LV mass, g/m^2^	0.01 (− 0.004 to 0.018)	0.001	− 0.02 (− 0.05 to 0.002)	0.07
T2D	0.67 (0.16 to 1.17)	0.01	1.49 (− 0.56 to 3.54)	0.15
BMI, kg/m^2^	− 0.06 (− 0.02 to 0.11)	0.004	0.03 (− 0.15 to 0.22)	0.72
eGFR, mL/min/1.73 m^2^	− 0.02 (− 0.03 to 0.002)	0.04	− 0.06 (− 0.13 to 0.002)	0.06
Urinary ACR, mg/g	0.0006 (0.0005 to 0.0008)	< 0.0001	0.001 (0.0006 to 0.002)	0.004
Urinary RBP4, mg/g	0.05 (0.02 to 0.07)	< 0.0001	0.15 (0.05 to 0.25)	0.003
FE phosphate, %	0.004 (− 0.01 to 0.02)	0.43	0.05 (− 0.03 to 0.12)	0.19
Use of RAS blockers	− 0.76 (− 1.28 to − 0.24)	0.004	− 0.55 (− 2.74 to 1.63)	0.62

	Multivariate			
	(log) uDPP4		sDPP4 (nmol/mL/min)	
	n = 426		n = 426	
	β (95% CI)	p-value	β (95% CI)	p-value
Age, years	0.002 (− 0.02 to 0.02)	0.83	− 0.29 (− 0.38 to − 0.20)	< 0.0001
Male sex	− 0.43 (− 0.85 to 0.06)	0.17	− 3.43 (− 5.49 to − 1.37)	< 0.0001
SBP, mmHg	0.003 (− 0.005 to 0.01)	0.40	0.03 (− 0.02 to 0.10)	0.16
LV mass, g/m^2^	0.009 (0.002 to 0.01)	0.01	− 0.008 (− 0.04 to 0.020)	0.56
T2D	0.57 (0.08 to 1.06)	0.02	1.31 (− 0.70 to 3.31)	0.21
BMI, kg/m^2^	− 0.07 (− 0.12 to 0.029)	0.001	− 0.12 (− 0.30 to 0.05)	0.17
eGFR, mL/min/1.73 m^2^	0.002 (− 0.01 to 0.02)	0.46	− 0.08 (− 0.15 to − 0.003)	0.08
Urinary ACR, mg/g	0.0006 (0.0004 to 0.0007)	< 0.001	0.0004 (− 0.0002 to 0.0015)	0.14
Urinary RBP4, mg/g	0.02 (− 0.004 to 0.05)	0.04	− 0.05 (− 0.18 to − 0.08)	0.47
Use of RAS blockers	− 0.52 (− 1.02 to 0.01)	0.03	0.14 (− 1.99 to 2.27)	0.88

Variables included in the multivariable-adjusted model: age, sex, T2D, BMI, SBP, eGFR, albuminuria, and RAS blockers. Data available for: LV mass, *n* = 412; BMI, *n* = 425; uRBP4, *n* = 415; FE Pi, *n* = 388; use of RAS blockers, *n* = 411.

*ACR* albumin creatinine ratio, *BMI* body mass index, *eGFR* estimated glomerular filtration rate, *LV* left ventricle, *RAS* renin-angiotensin system, *RBP4* retinol-binding protein 4, *SBP* systolic blood pressure, *T2D* type 2 diabetes.

### uDPP4 and sDPP4 and events of mortality and KRT

Among the 426 participants included in the cross-sectional analysis, vital status during follow-up was unavailable for 3 (0.7%), and KRT information was missing for 6 (1.4%). Over the 84-month follow-up period, there were 59 events of KRT and 181 deaths. Of the 59 participants who underwent KRT, 32 subsequently died. Plots illustrating the probability of remaining event-free, initiating KRT, or experiencing death as first events across tertiles of uDPP4 and sDPP4 activities over time are presented in Supplementary Fig. S3.

**Fig. 1:**
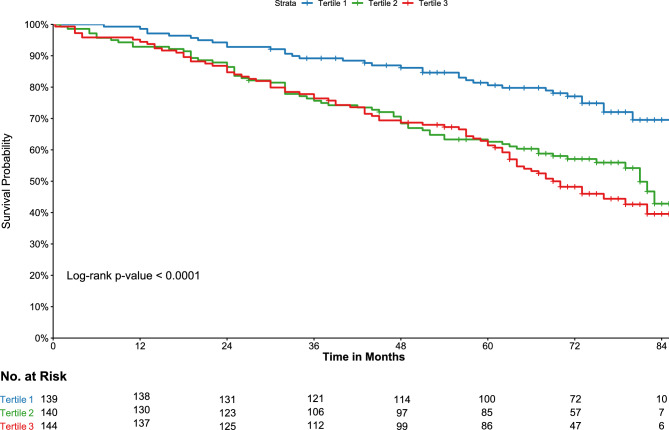
Kaplan–Meier survival curves for all-cause mortality according to the tertiles of urinary dipeptidyl peptidase 4 (uDPP4) activity.

**Fig. 2:**
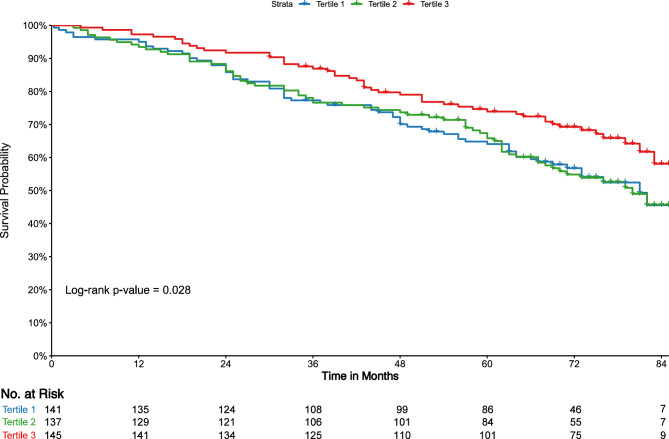
Kaplan–Meier survival curves for all-cause mortality according to the tertiles of serum dipeptidyl peptidase 4 (sDPP4) activity.

Survival curves across the tertiles of uDPP4 and sDPP4 are depicted in Figs. 1 and 2, respectively. Increased uDPP4 activity was associated with higher incidence rates of all-cause mortality (log-rank p < 0.001), as shown in Fig. 1. Conversely, lower levels of sDPP4 activity were associated with a higher incidence of death (log-rank p = 0.028) (Fig. 2). Tables 3 and 4 display the univariable and multivariable Cox models for mortality and KRT events, respectively. When analyzed as a continuous variable, higher log-transformed uDPP4 activity was associated with increased mortality risk. Notably, in the tertile analysis, participants in the second and third tertiles had higher mortality compared to those in the first tertile (HR 2.03, 95% CI 1.36–3.04 and 2.48, 95% CI 1.67–3.67 for T2 and T3, respectively), even after adjustment for potential confounders (Table 3). No association was observed between continuous sDPP4 activity and all-cause mortality. In the tertile-based analysis, participants in the lowest tertile of sDPP4 exhibited an increased mortality risk compared to those in the highest tertile; however, this association was not statistically significant after adjustment for confounders.

**Table 3:** Association between uDPP4 and sDPP4 activities with mortality.

	Unadjusted		Model 1		Model 2		Model 3		Model 4	
	HR (95% CI)	p-value	HR (95% CI)	p-value	HR (95% CI)	p-value	HR (95% CI)	p-value	HR (95% CI)	p-value
uDPP4 continuous (per 1 unit increase log 2)	1.18 (1.11–1.25)	< 0.0001	1.13 (1.07–1.18)	< 0.001	1.10 (1.05–1.16)	< 0.001	1.12 (1.07–1.18)	< 0.001	1.11 (1.06–1.17)	< 0.001
uDPP4 categorical										
Tertile 2 × Tertile 1	2.03 (1.36–3.04)	0.001	1.99 (1.32–2.99)	< 0.001	1.82 (1.20–2.76)	0.005	2.20 (1.45–3.36)	< 0.001	2.20 (1.41–3.42)	< 0.001
Tertile 3 × Tertile 1	2.48 (1.67–3.67)	< 0.0001	2.28 (1.52–3.42)	< 0.001	1.95 (1.28–2.98)	0.002	2.33 (1.52–3.55)	< 0.001	2.19 (1.40–3.41)	< 0.001
sDPP4 continuous	0.99 (0.97–1.00)	0.069	0.99 (0.98–1.01)	0.38	0.99 (0.98–1.01)	0.35	0.99 (0.98–1.01)	0.39	1.00 (0.98–1.01)	0.79
sDPP4 categorical										
Tertile 2 × Tertile 1	1.02 (0.72–1.44)	0.90	1.12 (0.79–1.60)	0.52	1.02 (0.72–1.45)	0.91	1.08 (0.76–1.55)	0.67	1.31 (0.89–1.91)	0.17
Tertile 3 × Tertile 1	0.65 (0.45–0.94)	0.023	0.75 (0.51–1.12)	0.16	0.74 (0.50–1.10)	0.14	0.76 (0.51–1.14)	0.18	0.87 (0.57–1.35)	0.54

Model 1: Adjusted for age, sex, type 2 diabetes (T2D), systolic blood pressure (SBP), and body mass index (BMI). Data available for 422 participants.

Model 2: Adjusted for age, sex, T2D, SBP, BMI, eGFR, and albuminuria. Data available for 422 participants.

Model 3: Adjusted for age, sex, T2D, SBP, BMI, eGFR, use of RAS blockers, and urinary retinol-binding protein 4 (uRBP4). Data available for 411 participants.

Model 4: Adjusted for age, sex, T2D, SBP, BMI, eGFR, use of RAS blockers, uRBP4, LDL, total cholesterol, LV mass, acute myocardial infarction, ejection fraction, and smoking. Data available for 389 participants.

**Table 4:** Association between uDPP4 and sDPP4 activities with KRT events using cause-specific hazard models.

	Unadjusted		Model 1		Model 2		Model 3		Model 4	
	HR (95% CI)	p-value	aHR (95% CI)	p-value	aHR (95% CI)	p-value	aHR (95% CI)	p-value	aHR (95% CI)	p-value
uDPP4 continuous (per 1 unit increase log 2)	1.10 (1.02–1.20)	0.018	1.08 (0.99–1.17)	0.075	0.98 (0.90–1.07)	0.70	1.06 (0.97–1.15)	0.18	1.06 (0.98–1.16)	0.17
uDPP4 categorical										
Tertile 2 × Tertile 1	1.19 (0.60–2.35)	0.62	1.17 (0.59–2.32)	0.66	0.94 (0.46–1.91)	0.86	1.18 (0.58–2.39)	0.65	1.11 (0.53–2.29)	0.79
Tertile 3 × Tertile 1	1.64 (0.86–3.10)	0.13	1.35 (0.69–2.63)	0.38	0.70 (1.28–2.98)	0.34	1.27 (0.64–2.51)	0.50	1.27 (0.63–2.59)	0.50
sDPP4 continuous	1.03 (1.01–1.05)	0.006	1.01 (0.99–1.04)	0.33	1.00 (0.98–1.02)	0.72	0.99 (0.97–1.01)	0.43	0.99 (0.96–1.01)	0.34
sDPP4 categorical										
Tertile 2 × Tertile 1	1.46 (0.72–2.98)	0.29	1.25 (0.60–2.58)	0.55	0.79 (0.37–1.67)	0.53	1.07 (0.48–2.35)	0.87	1.06 (0.46–2.44)	0.89
Tertile 3 × Tertile 1	1.80 (0.92–3.52)	0.086	1.24 (0.61–2.51)	0.55	1.02 (0.50–2.08)	0.96	1.01 (0.49–2.07)	0.98	1.07 (0.49–2.34)	0.86

Model 1: Adjusted for age, sex, type 2 diabetes (T2D), systolic blood pressure (SBP), and body mass index (BMI). Data available for 419 participants.

Model 2: Adjusted for age, sex, T2D, SBP, BMI, eGFR, and albuminuria. Data available for 419 participants.

Model 3: Adjusted for age, sex, T2D, SBP, BMI, eGFR, use of RAS blockers, and urinary retinol-binding protein 4 (uRBP4). Data available for 408 participants.

Model 4: Adjusted for age, sex, T2D, SBP, BMI, eGFR, use of RAS blockers, uRBP4, LDL, total cholesterol, LV mass, acute myocardial infarction, ejection fraction, and smoking. Data available for 386 participants.

*aHR* adjusted hazard ratio, *sDPP4* serum dipeptidyl peptidase 4, *uDPP4* urinary dipeptidyl peptidase 4.

Neither uDPP4 nor sDPP4 activity was associated with KRT initiation, whether analyzed as continuous variables or by tertiles, in adjusted cause-specific hazard models. These findings were consistent in Fine–Gray models using uDPP4 and sDPP4 tertiles (Supplementary Table S1). In contrast, participants in the second and third uDPP4 tertiles remained independently associated with the composite outcome of KRT or all-cause mortality after adjustment for prognostic variables (Supplementary Table S2).

## Discussion

We have previously demonstrated that uDPP4 activity, rather than sDPP4, increases during experimental kidney disease^20^. In this CKD cohort, we found that uDPP4 activity was independently associated with key markers of CKD and CVD, including elevated albuminuria, uRBP4, and LV mass. Additionally, individuals with CKD and elevated uDPP4 activity had a higher risk of all-cause mortality, even after adjustment for established risk factors.

In the cross-sectional analysis at baseline, high uDPP4 activity was associated with higher urinary levels of RBP4 and albuminuria, increasing progressively from the lower to the higher uDPP4 activity tertiles. These findings are in agreement with other evidence of increased uDPP4 activity in type 1 and type 2 diabetic nephropathy in association with microalbuminuria^29^.

In the prospective analysis, we observed an increased mortality risk among individuals with elevated uDPP4 levels, even after controlling for established prognostic factors in patients with CKD. However, we found no significant association between higher uDPP4 activity and the initiation of KRT. In CKD patients not on dialysis, all-cause mortality escalates with decreasing eGFR and increased albuminuria, independently of other risk factors^30^. Additionally, in patients with eGFR below 60 mL/min/1.73 m^2^, the risk of developing CVD surpasses the risk of KRT initiation^31^. Consistent with prior research, all-cause mortality in our cohort exceeded the incidence of KRT. Therefore, it is plausible that the low number of KRT cases in our cohort limits the statistical power for exploring this relationship, even considering the competing risk of death, as we observed nearly three times more terminal than KRT events in our cohort. Although an association between uDPP4 activity and KRT was initially observed, it did not persist after adjusting for known confounders.

Unlike sDPP4, which is known to originate from adipocytes^32^, endothelial cells^32^, and bone marrow^33^, the origin and biological significance of uDPP4 remain largely unexplored. sDPP4 is a high-molecular-weight protein that typically cannot pass through the glomerular filtration barrier under physiological circumstances. However, structural and functional changes to the glomerular barrier, such as those observed in CKD and albuminuria, may allow the passage of sDPP4, leading to its presence in the urine. On the other hand, the kidney is the organ with the highest levels of DPP4 in the body^2^, and renal DPP4 expression is upregulated in response to inflammatory stimuli. Moreover, our study demonstrates that the Pearson correlation between uDPP4 and sDPP4 is extremely weak (0.12). These findings lead us to hypothesize that uDPP4 may originate from increased shedding in kidney cells, specifically proximal tubular and/or glomeruli cells, in response to the inflammatory milieu of CKD and albuminuria. This hypothesis is further supported by reports of glucose-induced DPP4 shedding by human glomerular cells^34^ and increased urinary DPP4-positive vesicles in patients with T2D compared to healthy individuals^35^. Notably, T2D individuals in our cohort exhibit significantly higher uDPP4 activity than nondiabetic participants, while sDPP4 activity does not differ between these groups (Supplementary Fig. S4A,B).

A potential upstream regulator of DPP4 shedding and activity in CKD is the renin-angiotensin II system (RAS). Studies have shown that RAS signaling enhances the expression of enzymes involved in DPP4 shedding^36^, and infusion of supraphysiological concentrations of angiotensin II (Ang II) increase kidney DPP4 activity^37^. Ang II treatment of cultured proximal tubule cells classically activates the mitogen-activated protein kinase (MAPK)—extracellular signal-regulated kinase 1/2 (ERK 1/2) pathway, consequently elevating DPP4 activity^37^. Furthermore, concurrent DPP4 inhibition during Ang II treatment attenuates MAPK-ERK 1/2 activation^38^, illustrating a bidirectional interaction between the signaling mechanisms triggered by Ang II and DPP4^39^. In the 5/6 nephrectomy model of kidney disease, restoration of megalin expression and amelioration of tubular and glomerular proteinuria by DPP4 inhibition was accompanied by suppression of the kidney disease-associated upregulation of renal Ang II^20^. Consistently, Nistala et al. found that DPP4 inhibition mitigates Ang II-induced kidney injury and albuminuria^40^. Additionally, our current study reveals that uDPP4 activity inversely correlates with the use of RAS blockers. Moreover, participants on these medications exhibit significantly reduced uDPP4 activity, yet these blockers do not affect sDPP4 activity (Supplementary Fig. S4C,D). Thus, we propose that RAS signaling prompts an increased DPP4 shedding and activity, acting as a feed-forwarding mechanism for RAS signaling-mediated inflammation and tissue damage during CKD. It is known that sDPP4 acts as a costimulatory molecule for inflammatory cells^41^ and that DPP4 expression in fibroblasts accompanies collagen upregulation^42^. In agreement with these roles, DPP4 inhibition blunts apoptosis, inflammatory cell infiltration, oxidative stress, glomerular hypertrophy, and renal fibrosis^43,44^.

In contrast to uDPP4 activity, sDPP4 activity was not associated with the variables related to CKD. Our data argue against the evidence of Cho et al., who found a correlation between sDPP4, creatinine, and GFR in diabetic patients^45^. Previous research has also observed an association between sDPP4 activity and BMI, which was not reproduced in this analysis^15^. Importantly, these discrepancies could be attributed to the earlier studies measuring sDPP4 abundance rather than activity, which do not necessarily correlate^32^. Indeed, sDPP4 abundance is known to be induced by systemic DPP4 enzymatic inhibition^32^. Consistent with previous research conducted by our laboratory^16,19^ and others^46,47^, our results demonstrate an inverse association between sDPP4 activity and age. One potential biological implication of reduced sDPP4 activity with age may be linked to the age-associated decline in appetite. In support of this, a meta-analysis^48^ and a pilot study^49^ have reported that circulating concentrations of appetite-regulating hormones, including DPP4 substrates such as PYY and GLP-1, are higher in healthy older adults than younger adults.

This study has several limitations. First, the modest sample size may have limited our ability to detect small differences between groups. Second, the higher number of deaths (n = 181) compared to KRT events (n = 59) may have reduced the power to detect an association between uDPP4 and KRT outcomes. Third, although uDPP4 was normalized to urinary creatinine concentration, the accuracy of this approach may be reduced in CKD, where creatinine handling by the kidney, including filtration and tubular secretion, can be altered. Fourth, because sDPP4 and uDPP4 activities, as well as other kidney markers, were measured only at baseline and not during follow-up, we could not assess their association with surrogate biomarkers of CKD progression or the diagnostic value of longitudinal changes in DPP4 activity. Finally, unmeasured confounding cannot be excluded from the observed association between uDPP4 activity and all-cause mortality.

In synthesis, we provided evidence that uDPP4, but not sDPP4, is associated with an increased risk of all-cause mortality in people with CKD. Additionally, higher uDPP4 activity was linked to markers of renal dysfunction, such as albuminuria and urinary retinol-binding protein 4, which may reflect ongoing kidney injury and function decline. These findings align with previous implications of uDPP4 activity in the development and worsening of experimental CKD and shed light on potential new pathophysiological mechanisms for human CKD.

## Methods

### Study design and participants

The PROGREDIR cohort included 454 CKD participants from the outpatient service of Hospital das Clinicas in Sao Paulo, Brazil^48,49^. In brief, adult individuals being followed up at the outpatient setting with at least two elevated serum creatinine concentrations (≥ 1.6 mg/dL for men and ≥ 1.4 mg/dL for women) collected at least 90 days apart were invited to participate in the study. Key exclusion criteria were recent hospitalization or acute myocardial infarction, acute or active glomerulonephritis, current use of immunosuppressive drugs or chemotherapy, ongoing dialysis, hepatitis B, hepatitis C, HIV, severe mental illness, pregnancy, and participation in another clinical trial. Additionally, participants using DPP4 inhibitors (n = 2) or those lacking sufficient urine samples (n = 26) were excluded from this analysis. All participants provided written informed consent. Further details about the PROGREDIR cohort can be found elsewhere^48,49^.

The study procedures comprised early morning fasting blood tests, single-sample and 24-h urinary tests, medical history assessments, and anthropometric evaluations. Blood pressure (BP) was measured using the average of two readings taken after an initial measure with an oscillometer (Omron HEM 705CPINT). Diabetes was defined by a combination of self-reported history, medication use, fasting plasma glucose ≥ 126 mg/dL, glycated hemoglobin ≥ 6.5%, or two-hour plasma glucose ≥ 200 mg/dL. Hypertension was defined by self-reported history. Creatinine was measured by colorimetric assay and standardized to reference material. The estimated glomerular filtration rate (eGFR) was calculated using the 2021 CKD-EPI creatinine equation^50^. As previously described, albuminuria and urinary retinol-binding protein 4 (RBP4) were measured in spot urine samples and normalized by urinary creatinine^48,49^. All blood and urine samples were aliquoted and cryopreserved for analysis. Transthoracic echocardiography was performed in all participants using an Aplio XG device (Toshiba Corporation, Tokyo, Japan) with a 2.5 Hz sector transducer. The same echocardiographer conducted all examinations. Left ventricle (LV) mass was calculated using the Devereaux formula^51^ and adjusted for body surface area.

Annual follow-up involved telephone interviews to gather data on mortality, hospitalizations, and the necessity for kidney replacement therapy (KRT). Vital status was periodically verified using a “hot pursuit” strategy^52^, with mortality data validated through official death certificates and cooperation with health offices (PRO-AIM, Fundação SEADE, and the Brazilian National Mortality Registry). KRT tracking was managed via the São Paulo State and City Registries of Dialysis and Kidney Transplantation. The study received approval from the Ethics Committee on Human Research at the Hospital Universitario of the University of Sao Paulo (no. 11147/11) and the Ethics Committee for Analysis of Research Projects at the Hospital das Clinicas, HCFMUSP (no. 0798/11). All methods were carried out in accordance with the relevant guidelines and regulations.

### DPP4 activity assay

Serum and urinary DPP4 (sDPP4 and uDPP4, respectively) activities were measured using a modified colorimetric method based on the protocol described by Hopsu-Havu^53^. This method employs the synthetic substrate glycyl-prolyl-p-nitroanilide tosylate (H-Gly-Pro-pNA-HCl, Bachem L-1880.1000), which releases p-nitroaniline upon hydrolysis by DPP4. For the assays, duplicate aliquots of 15 µL of serum or 200 µL of urine were incubated with 2 mM H-Gly-Pro-pNA-HCl in 10 mM Tris–HCl buffer (pH 8.0) at 37 °C. Absorbance at 450 nm was recorded every 10 min over a 60-min incubation period (SpectraMax M5, Molecular Devices) at an absorbance of 450 nm. Experiments were conducted in the presence or absence of the selective DPP4 inhibitor sitagliptin (10 µM). DPP4 activity was calculated based on a standard curve of the substrate p-nitroaniline, with results expressed in nmol/mL/min. uDPP4 activity was further normalized to urinary creatinine and expressed as nmol/min/g.

### Statistical analysis

Categorical data are presented as counts and percentages, and continuous data are presented as either mean ± standard deviation (SD) or median with interquartile ranges (IQR), as appropriate. Participants were divided into tertiles based on uDPP4 and sDPP4 activities. Comparisons of continuous variables across DPP4 activity tertiles were performed using the Kruskal–Wallis test, with Tukey’s post-hoc test for normally distributed variables and Friedman’s test for non-normally distributed variables. The Chi-squared test was used to compare categorical variables. Due to high variability and right-skewed distribution, uDPP4 was log-transformed. Associations between uDPP4 and sDPP4 activities with age, biological sex, and renal, cardiovascular, and metabolic function markers were explored using univariable and multivariable linear regression models. Variables that showed significant associations with uDPP4 or sDPP4 based on Pearson’s correlation, along with clinically relevant factors, were included as confounders in the multivariable models.

Time-to-event analysis for all-cause mortality across tertiles of uDPP4 and sDPP4 activities was performed using Kaplan–Meier survival curves, with comparisons made using the log-rank test. Cox regression models were fitted separately for all-cause mortality and initiation of KRT using uDPP4 and sDPP4 activity as continuous variables and by tertiles, with the first tertile as the reference group. To account for the competing risk of death in KRT, we used cause-specific and Fine-Gray models^54^. The association between uDPP4 tertiles and the composite outcome of KRT initiation or all-cause mortality was also assessed using Cox regression models. All models were standardized across outcomes, with Models 1–4 applied uniformly and each maintaining an identical covariate structure. No imputation was performed. Statistical analyses were conducted using R version 4.3.2 or higher (R Foundation for Statistical Computing, Vienna, Austria). Figures were generated using R and GraphPad Prism 10 (GraphPad Software, San Diego, CA, USA).

## Supplementary Information

Supplementary Information.

## Data Availability

All data generated or analyzed during this study are included in this article. Further inquiries can be directed to the corresponding author.
