# Supplementary material for: Association of urinary dipeptidyl peptidase 4 activity with clinical outcomes in people with chronic kidney disease
Source: Sci Rep. 2025 Jul 2;15:23190. doi: 10.1038/s41598-025-06395-x (PMC12222852; doi:10.1038/s41598-025-06395-x)
Supplement: Supplementary file 1 — Supplementary Information. [file 41598_2025_6395_MOESM1_ESM.docx]

**ONLINE SUPPLEMENTAL DATA**

**Association of urinary dipeptidyl peptidase 4 activity with clinical outcomes in people with chronic kidney disease**

Acaris Benetti^1^, Joao Carlos Ribeiro-Silva^2^, Luz M. Gómez^3^, Caio A. M. Tavares^4,5^, Isabela J. Bensenor^6^, Paulo A. Lotufo^6^, Silvia M. O. Titan^7,8^, Adriana C. C. Girardi^1^

^1^Laboratório de Genética e Cardiologia Molecular, Instituto do Coração (InCor), Hospital das Clinicas HCFMUSP, Faculdade de Medicina, Universidade de São Paulo, São Paulo, SP, Brazil; ^2^State University of New York (SUNY) Upstate Medical University, Syracuse, NY, USA; ^3^Departamento de Estatistica, Universidade Federal de Pernambuco, Recife, PE, Brazil; ^4^Unidade de Geriatria, Instituto do Coração (InCor), Hospital das Clínicas HCFMUSP, Faculdade de Medicina, Universidade de São Paulo, São Paulo, SP, Brazil;  ^5^Hospital Israelita Albert Einstein, São Paulo, São Paulo, Brazil; ^6^Hospital Universitario, Universidade de Sao Paulo, Sao Paulo, Brazil. ^7^Divisão de Nefrologia, Hospital das Clínicas HCFMUSP, Faculdade de Medicina, Universidade de São Paulo, São Paulo, SP, Brazil; ^8^Nephrology and Hypertension Division, Mayo Clinic, Rochester, MN, USA.

**Table S1 - Association between uDPP4 and sDPP4 activities and initiation KRT using the Fine-Gray hazard models**

|  | **Unadjusted** | |  | **Model 1** | | | |  | **Model 2** | |  | **Model 3** | |  | **Model 4** | |
| --- | --- | --- | --- | --- | --- | --- | --- | --- | --- | --- | --- | --- | --- | --- | --- | --- |
|  | **HR (95% CI)** | **p-value** |  | **aHR (95% CI)** | | **p-value** | |  | **aHR (95% CI)** | **p-value** |  | **aHR (95% CI)** | **p-value** |  | **aHR (95% CI)** | **p-value** |
| **Fine-Gray Hazard Models**  **uDPP4 categorical** |  |  |  |  | |  | |  |  |  |  |  |  |  |  |  |
| **Tertile 2 x Tertile 1** | 1.04 (0.53 - 2.06) | 0.90 |  | 1.08 (0.54 - 2.13) | | 0.83 | |  | 0.89 (0.46-1.72) | 0.74 |  | 1.09 (0.54-2.21) | 0.81 |  | 1.07 (0.52-2.19) | 0.79 |
| **Tertile 3 x Tertile 1** | 1.41 (0.75 - 2.65) | 0.29 |  | 1.19 (0.61 - 2.32) | | 0.60 | |  | 0.53 (0.28-1.00) | 0.049 |  | 0.98 (0.47-2.04) | 0.95 |  | 1.07 (0.49-2.32) | 0.50 |
| **sDPP4 categorial** |  |  |  |  | |  | |  |  |  |  |  |  |  |  |  |
| **Tertile 2 x Tertile 1** | 1.48 (0.73 - 3.00) | 0.28 |  | 1.25 (0.60 - 2.61) | | | 0.54 |  | 0.76 (0.37 - 1.55) | 0.45 |  | 0.95 (0.42 - 2.16) | 0.91 |  | 0.86 (0.36 - 2.05) | 0.74 |
| **Tertile 3 x Tertile 1** | 1.98 (1.02 - 3.86) | 0.045 |  | 1.32 (0.65 - 2.69) | 0.44 | | |  | 0.89 (0.44 - 1.77 | 0.73 |  | 0.99 (0.48 - 2.02) | 0.97 |  | 1.09 (0.51 - 2.35) | 0.82 |

Abbreviations: aHR, adjusted hazard ratio; sDPP4, serum dipeptidyl peptidase 4; uDPP4, urinary dipeptidyl peptidase 4.

Model 1: Adjusted for age, sex, type 2 diabetes (T2D), systolic blood pressure (SBP), and body mass index (BMI). Data available for 419 participants.

Model 2: Adjusted for age, sex, T2D, SBP, BMI, eGFR, and albuminuria. Data available for 419 participants.

Model 3: Adjusted for age, sex, T2D, SBP, BMI, eGFR, use of RAS blockers, and urinary retinol-binding protein 4 (uRBP4). Data available for 408 participants.

Model 4: Adjusted for age, sex, T2D, SBP, BMI, eGFR, use of RAS blockers, uRBP4, LDL, total cholesterol, LV mass, acute myocardial infarction, ejection fraction, and smoking. Data available for 386 participants

**Table S2 - Association between uDPP4 activity and the composite outcome of initiation KRT or death**

|  | **Unadjusted** | |  | **Model 1** | |  | **Model 2** | |  | **Model 3** | |  | **Model 4** | |
| --- | --- | --- | --- | --- | --- | --- | --- | --- | --- | --- | --- | --- | --- | --- |
|  | **HR (95% CI)** | **p-value** |  | **aHR (95% CI)** | **p-value** |  | **aHR (95% CI)** | **p-value** |  | **aHR (95% CI)** | **p-value** |  | **aHR (95% CI)** | **p-value** |
|  |  |  |  |  |  |  |  |  |  |  |  |  |  |  |
| **uDPP4 categorical** |  |  |  |  |  |  |  |  |  |  |  |  |  |  |
| **Tertile 2 x Tertile 1** | 1.82 (1.25 - 2.64) | 0.002 |  | 1.72 (1.18 - 2.50) | 0.005 |  | 1.56 (1.06 - 2.28) | 0.023 |  | 1.94 (1.32 - 2.84) | <0.001 |  | 1.60 (1.06 - 2.39) | 0.024 |
| **Tertile 3 x Tertile 1** | 2.32 (1.62 - 3.33) | <0.001 |  | 2.07 (1.43 - 2.99) | <0.001 |  | 1.67 (1.14 – 2.45) | 0.008 |  | 2.09 (1.43 – 3.06) | 0.005 |  | 1.58 (1.05 – 2.39) | 0.027 |
|  |  |  |  |  | |  |  |  |  |  |  |  |  |  |

Abbreviations: aHR, adjusted hazard ratio; uDPP4, urinary dipeptidyl peptidase 4.

Model 1: Adjusted for age, sex, type 2 diabetes (T2D), systolic blood pressure (SBP), and body mass index (BMI). Data available for 419 participants.

Model 2: Adjusted for age, sex, T2D, SBP, BMI, eGFR, and albuminuria. Data available for 419 participants.

Model 3: Adjusted for age, sex, T2D, SBP, BMI, eGFR, use of RAS blockers, and urinary retinol-binding protein 4 (uRBP4). Data available for 408 participants.

Model 4: Adjusted for age, sex, T2D, SBP, BMI, eGFR, use of RAS blockers, uRBP4, LDL, total cholesterol, LV mass, acute myocardial infarction, ejection fraction, and smoking. Data available for 386 participants

**Figure S1**

**
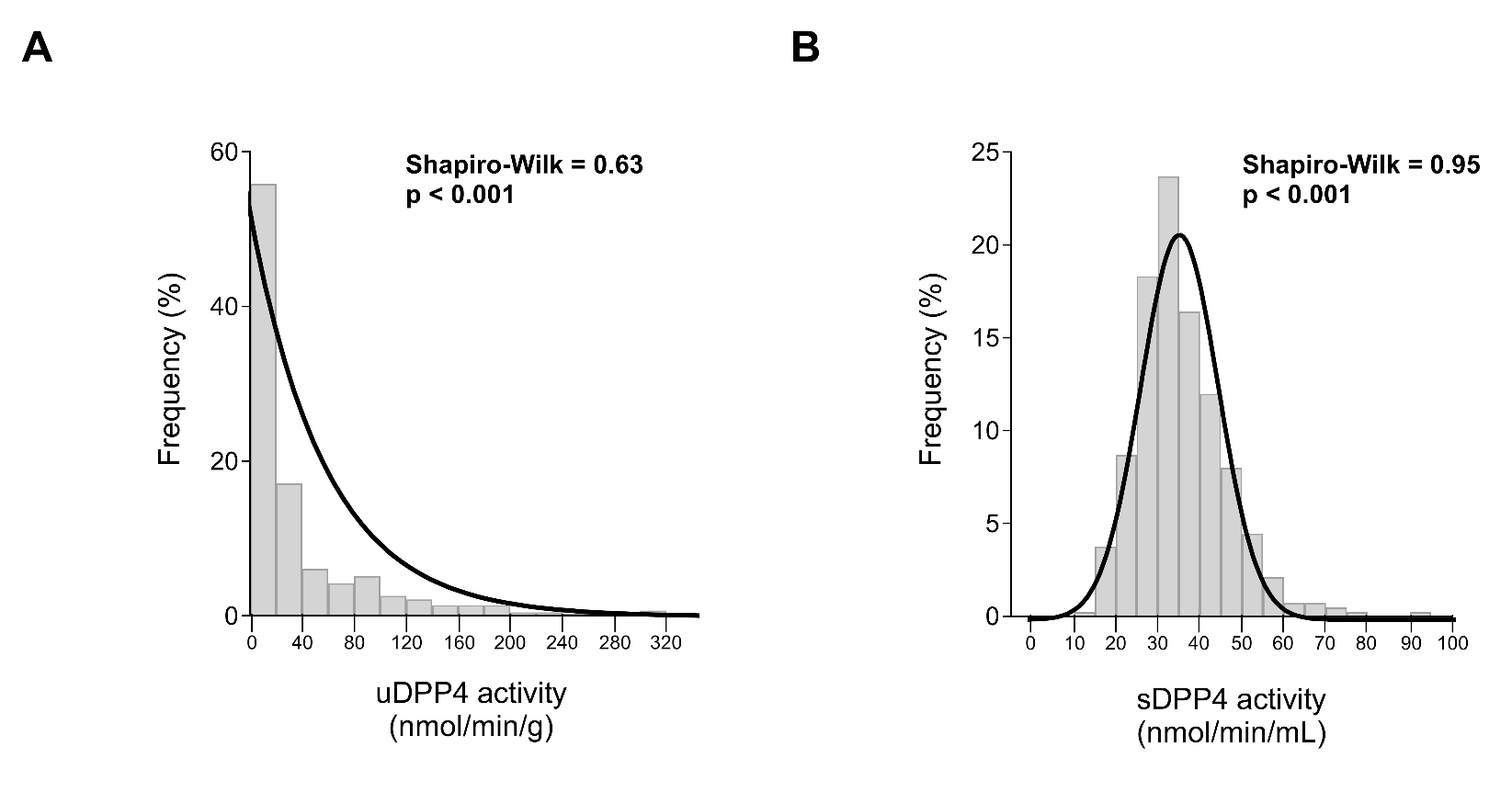
**

**Figure S1 – Frequency distribution of urinary and serum DPP4 activity among participants in the PROGREDIR cohort.** **(A)** Histogram showing the distribution of urinary DPP4 (uDPP4) activity, expressed as nmol/min/g of urinary creatinine. The data exhibit a right-skewed distribution, and the Shapiro-Wilk test confirmed a significant deviation from normality (W = 0.63, p < 0.001). **(B)** The histogram of serum DPP4 (sDPP4) activity is expressed in nmol/min/mL. The distribution approximates normality but still deviates significantly, as indicated by the Shapiro-Wilk test (W = 0.95, p < 0.001). In both panels, the solid black line represents the fitted distribution curve for visual assessment of normality.

**Figure S2**

**
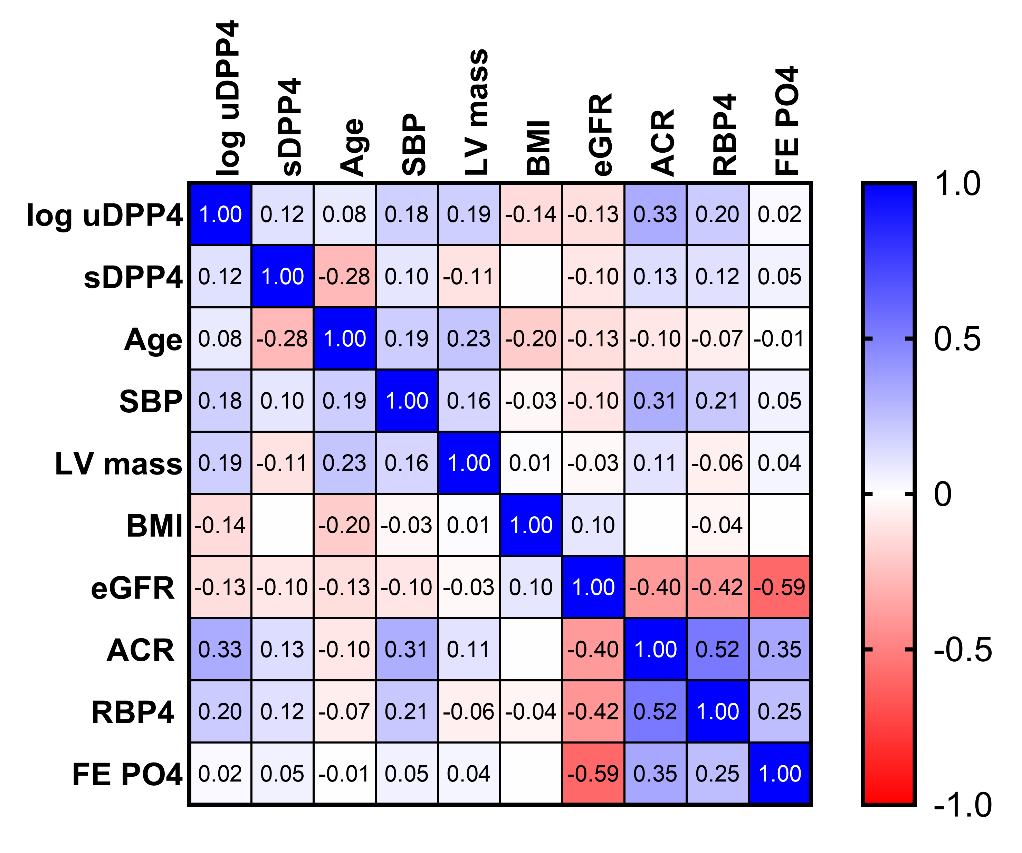
**

**p-Values**

|  | **log uDPP4** | **sDPP4** | **Age** | **SBP** | **LV mass** | **BMI** | **eGFR** | **ACR** | **RBP4** | **FE PO_4_** |
| --- | --- | --- | --- | --- | --- | --- | --- | --- | --- | --- |
| **log uDPP4** |  | 0.007 | 0.05 | <0.0001 | <0.0001 | 0.002 | 0.004 | <0.0001 | <0.0001 | 0.33 |
| **sDPP4** | 0.007 |  | <0.0001 | 0.02 | 0.01 | 0.49 | 0.02 | 0.003 | 0.009 | 0.17 |
| **Age** | 0.05 | <0.0001 |  | <0.0001 | <0.0001 | <0.0001 | 0.004 | 0.03 | 0.08 | 0.45 |
| **SBP** | <0.0001 | 0.02 | <0.0001 |  | <0.001 | 0.25 | 0.02 | <0.0001 | <0.0001 | 0.15 |
| **DBP** | 0.43 | 0.003 | <0.0001 | <0.0001 | 0.48 | 0.01 | 0.03 | <0.0001 | 0.01 | 0.28 |
| **LV mass** | <0.0001 | 0.01 | <0.0001 | <0.001 |  | 0.43 | 0.27 | 0.01 | 0.10 | 0.24 |
| **BMI** | 0.002 | 0.49 | <0.0001 | 0.25 | 0.43 |  | 0.02 | 0.47 | 0.19 | 0.48 |
| **eGFR** | 0.004 | 0.02 | 0.004 | 0.02 | 0.27 | 0.02 |  | <0.0001 | <0.0001 | <0.0001 |
| **ACR** | <0.0001 | 0.003 | 0.02 | <0.0001 | 0.01 | 0.47 | <0.0001 |  | <0.0001 | <0.0001 |
| **RBP4** | <0.0001 | 0.009 | 0.08 | <0.0001 | 0.10 | 0.19 | <0.0001 | <0.0001 |  | <0.0001 |
| **FE PO_4_** | 0.33 | 0.17 | 0.45 | 0.15 | 0.24 | 0.48 | <0.0001 | <0.0001 | <0.0001 |  |
|  |  |  |  |  |  |  |  |  |  |  |

**Figure S2 – Spearman correlation coefficients and p-values for log-transformed uDPP4 activity, sDPP4 activity, and continuous variables related to demographics and renal function.** Blue indicates a positive correlation, while red indicates a negative correlation between variables. Darker colors represent stronger correlations. The variables were assessed in the following units: uDPP4 (log-transformed, nmol/min/g); sDPP4 (nmol/mL/min); age (years); body mass index (BMI, kg/m²); estimated glomerular filtration rate (eGFR, mL/min/1.73 m²); urinary albumin-to-creatinine ratio (ACR, mg/g); urinary retinol-binding protein 4 (uRBP4, mg/g); fractional excretion of phosphate (FE PO₄, %); and systolic blood pressure (SBP, mmHg).

**Figure S3**

**A**


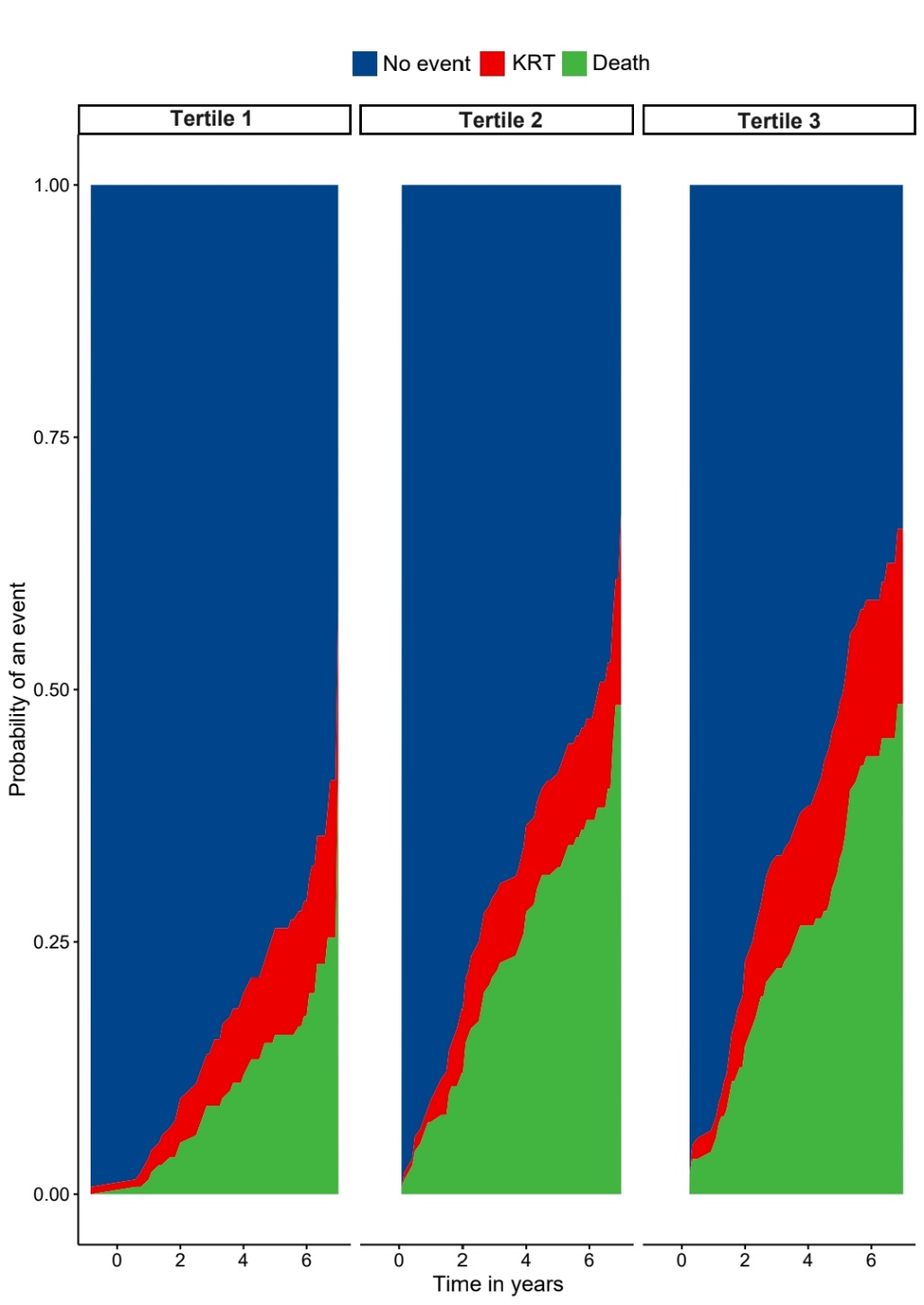


**B**


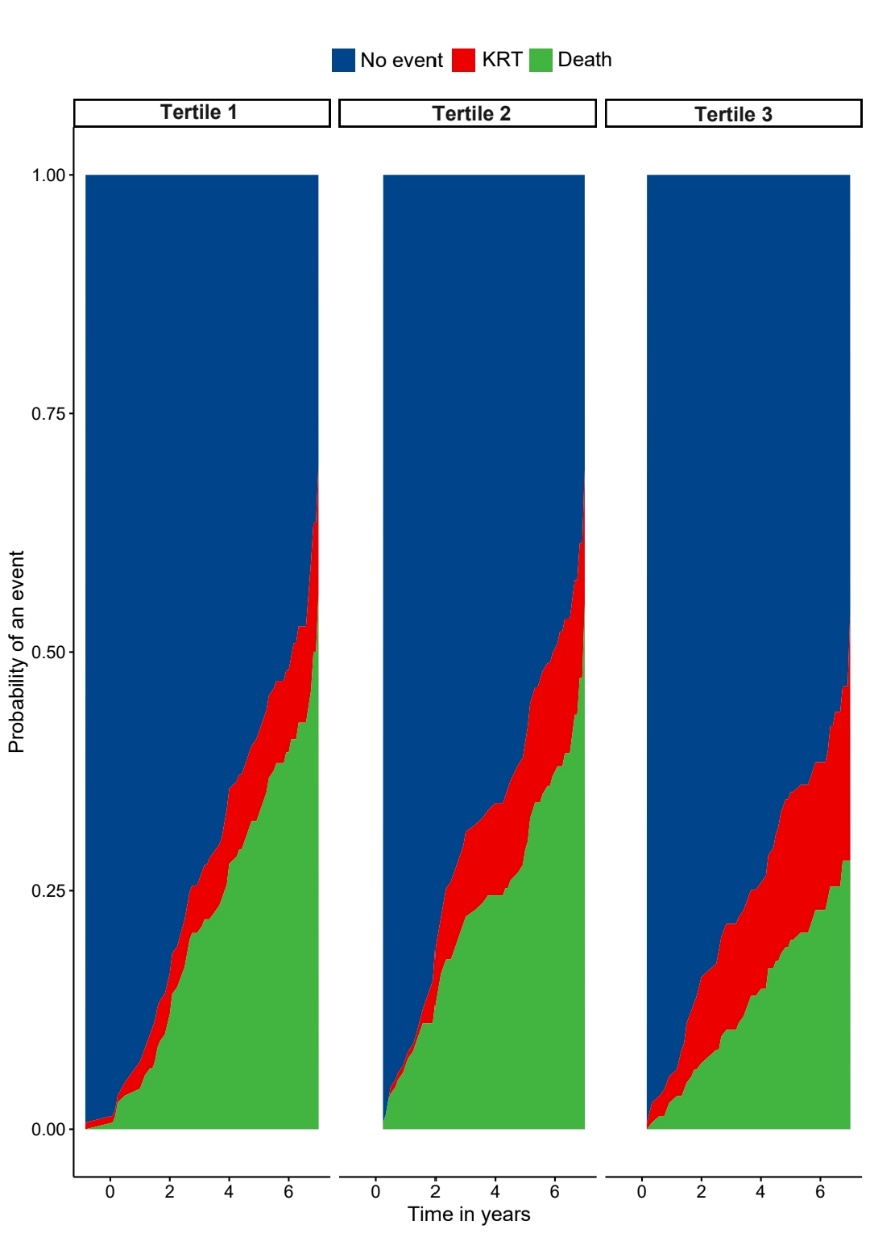


**Figure S3 – Probability of remaining event-free, initiating kidney replacement therapy (KRT), or death as first events according to tertiles of DPP4 activity in participants from the PROGREDIR cohort. (A)** Tertiles of urinary DPP4 (uDPP4) activity, expressed in nmol/min/g of urinary creatinine. **(B)** Tertiles of serum DPP4 (sDPP4) activity, expressed in nmol/min/mL. Stacked probability plots show the likelihood of being in each state (event-free, KRT as first event, or death as first event) over time (in years) across tertiles of DPP4 activity. The y-axis represents the probability of being in each state, while the x-axis represents follow-up time in yea

**Figure S4**


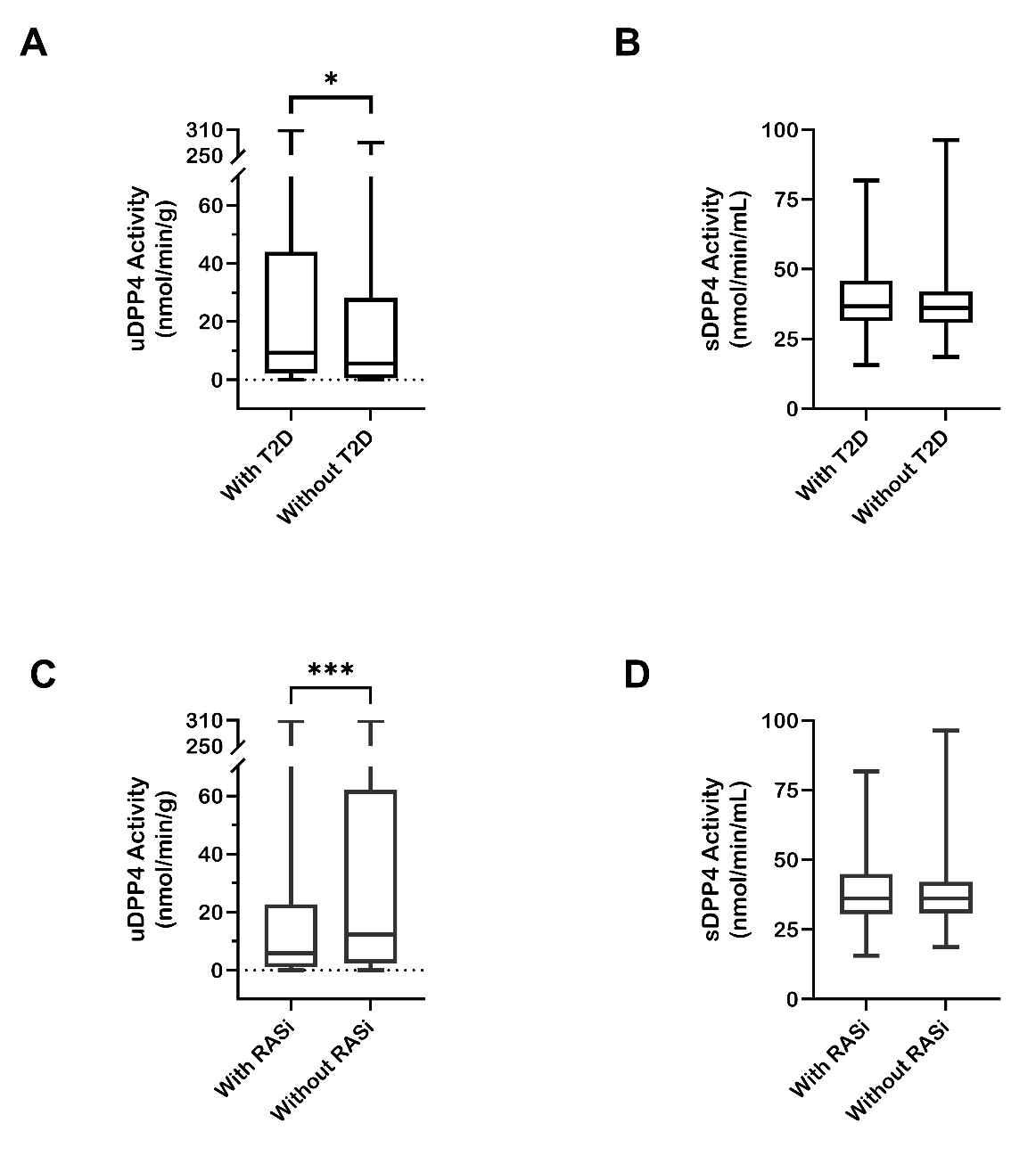


**Figure S4 – Urinary and serum DPP4 activity levels according to type 2 diabetes (T2D) status and use of renin-angiotensin system blockers (RASi) in participants from the PROGREDIR cohort. (A)** uDPP4 activity (nmol/min/g creatinine) was significantly higher in participants with T2D compared to those without T2D (*p < 0.05, Mann-Whitney test). **(B)** sDPP4 activity (nmol/min/mL) did not significantly differ between participants with and without T2D. **(C)** uDPP4 activity was significantly lower in individuals using RAS blockers compared to those not using RASi (**p < 0.001, Mann-Whitney test). **(D)** No significant difference in sDPP4 activity was observed between participants based on RASi use. Boxes represent the interquartile range (IQR), and horizontal lines denote the median.
